# The Newest of Our Creeds

**Published:** 1881-12

**Authors:** 


### ARTICLE II.

### *The Newest of Our Creeds.*

BY PROF. HODGKIN.

The Dental Profession is not prolific in book-making. Half a dozen on our side of the water and less perhaps than that number over in the old world, make up the recorded sum of what has been written, outside of the chronic shower of strong and weak periodic literature which is continually rained through the various journals which, monthly and quarterly appear through the mails; so that a new book is something of a sensation. And one has appeared. Dr. J. Foster Flagg, who must surely be one of the most industrious of mortals, since, in addition to caring for the teeth of sundry thousands of families, contributes sprightly papers to the journals, reads papers before dental societies, and teaches in a dental college, has lately brought out a very new book of 200 pages on "Plastics and Plastic Fllings;" published by Presley Blackiston, 1012 Walnut Street, Philadelphia, in handsome style, fine tinted paper, large type, and written in a style which is eminently like Dr. Flagg. In the preface the author states as an apology "for some of the peculiarities of composition" that the "articles" were originally intended for magazine contributions, but he concluded to offer them in book form for the good, as he hopes and intends, of the profession. The title page is "Plastics and plastic filling; as pertaining to the filling of all cavities of decay in teeth below medium in structure, and to difficult and inaccessible cavities in teeth of all grades and structure."

Manifestly a book or a man who runs athwart the traditions of his generation will attract attention and denunciation, and it is fair to presume and predict in advance that this book will be largely read, and doubtless largely influence professional opinion. But whatever else the reader may think of this book he can come to but one conclusion regarding one feature of it. Dr. Flagg is very much in earnest, and has strong convictions although it would seem that these partake of the nature of his subject—are plastic—therecan be no doubt about that. There is in this book no halting or hesitating, but little quoting of authorities and leaving the reader to judge for himself of the truth of the doctrine quoted. Dr. Flagg is evidently a born fighter, and puts up as his motto: "Truth without fear or favor." How far those who read him will agree with him as to his *find-*

*ing* the truth, we may see. The wonder is that one man can do all the observing, "testing," experimenting, and "proving" that Dr. Flagg does, and find time meanwhile for the eating, drinking, sleeping, and daily work of attending to the teeth of the 5000 families whose teeth he is reported daily to save.

The old saying that "he who follows must always be behind," must we suppose, have a converse, and he who leads must always go before—in some cases a long way before indeed, and Dr. Flagg illustrates this. To use nothing but "plastics," for the sake of proving their usefulness; to discard gold altogether because it is not always best—Dr. F. thinks it is seldom best—to fill all roots with cotton and glycerine because all roots cannot be well and solidly filled with something else; to use one make of amalgam for the cervical wall and another for the body of the filling, and still another for tne "contour," and yet another for the facing, with linings of gutta-percha, linings oxy-chloride, etc.; to introduce a nomenclature of new terms, based on the new principles, is a "*new* departure" truly. Few, save Dr. Flagg would have vim enough for it. But he seems to have wonderful elasticity and spontaniety, jumping at the "new" with new freshness every time. Somewhere in the past he is on record, if memory serves the writer, as enthusiastic on the subject of somebody's plastic gold, with which he could fill a cavity in a minute or two at the most.

But we have no desire to pick flaws in the past record of Dr. Flagg, if he is right now—if!

For alas! teeth are lost still, even with Lawrence's, the Standard, gutta-percha and other fillings, never so carefully inserted by careful and conscientious men, and teeth, yes frail and *soft teeth are saved with gold*, for years and years! And so we fear it will be to the end. True it is, as Dr. Flagg most truly states, that the labor to the operator, of inserting a difficult gold filling and the endurance on the part of the patient at receiving such a filling, and the costliness of it all,—to operator in nervous strain and physical wear and tear; and to patient in exhaustion of system and finances—is indeed worth counting, and we feel it has never been put in so strong light before. Patients *do dread* the mallet and the rubber dam and all these appliances, and dread the long sitting with wide-stretched jaws, and some even dread the bill; and from this stand-point Dr. Flagg has a strong argument indeed, for *comfort* is worth attainment.

Dr. Flagg says it is twenty-five years now since he commenced the work of the "systematic development of plastic fillings," that it "has been a long and sometimes tedious path," and he found himself at the end of the ninth year filling all teeth below medium in structure, with plastic materials. He asserts that with "dentures selected for their frailty" he is able to "meet all the indications without a resort to artificial work," not including in this term "artificial" the operation of pivoting; and that "during the past eight years the requirements of all my patients have not reached an average of one piece of artificial work per annum." Of course this implies that the edentulous do not apply to Dr. Flagg; and as the public soon come to understaod that a dentist does not do artificial work, and those needing it naturally go elsewhere, at least so we explain what may otherwise seem a remarkable statement. True Dr. Flagg says he sought for the bad cases, but it need not follow that they all went to him, for we know how sadly people get discouraged about their teeth and decide "to let them go."

The early "history of amalgam" as given by Dr. Flagg is interesting in a high degree, and if the book has no other value it will be useful for this chapter alone. The conduct of the American Society of Dentists in deciding to expel its amalgam users, is an additional proof that they deserve place with the medical profession, which is on record as nobly in the past in matters still more important. But if Dr. Flagg is to be credited, the lack of knowledge on the part of those professing to be the scientific men and leaders of the profession is simply amazing. He digs deep into the journalistic literature of the subject, and brings forth a mass of contradictory statements, of errors of analysis, of jumbled guess-work that shews, if nothing more, that the students who were working in that line, had much to learn. It seems a reasonable and sensible act on the part of Dr. Flagg to get help of men who were professional essayrs; the reports they have made *should be* correct. In the paragraph, page 39, the words "the incentive to them, [the assayers of the U. S. Mint] was the probability of eventually ariving at an excellent alloy, which they could manufacture," probably furnished the key to the careful concealment of the formula of the "Standard alloy" which Dr. Flagg makes haste earnestly to assure his readers he has had pecuniary interest in. But it is a little annoying to find so many of these alloys duly set forth, and this as duly concealed. For one does like, if only for satisfaction sake, to know what one is using.

It is right hard on the amalgam *makers* to charge them with furnishing special samples for analysis to Prof. Wood; and if it be true that gold or platinum were added to samples furnished, for the *effect*, while these were wholly or or partly left out in the real manufacture, it argues singular wickedness, if these substances are useful in such alloys, though Dr. Flagg puts his foot down on platinum so strongly as to make the leaving of this metal out no disadvantage.

Lately Dr. T. O. Oliver has stepped into the field with a "new and independent" *Quarterly*, in which he insinuates that Dr. Flagg tried to get in an underhand manner the formula for *his* amalgam, and is quite sarcastic and severe on this book. Especially is the platinum presence strongly insisted upon as essential. How it came to pass that Dr. H. S. Chase, working as a co-worker with Dr. Flagg, puts out an alloy simply of tin and gold and commends it as "the best," while Dr. Flagg himself at the other end of the line does not mention this combination at all, but leaves his readers to infer that tin, in any large proportion, unless over-shadowed by silver in still larger proportion, is detrimental, and that only a small per cent. of gold is useful, is puzzling. He says "gold and platina alloys cannot be in much degree suporior to the ordinary alloys of tin and silver, and in name and inference of great superiority, these alloys are deceptive." If his conclusions are correct and if the makers of the alloys heralded as non-shrinking, etc., enter this fight the battle is not yet over.

A very ingenious theory given on page 43 in the chapter on "the attributes of metals and for amalgam alloys" is that the fusing points of metals is lowered by the mercury, causing softening of the alloy when brought into contact with this fluid metal, and that the subsequent "setting" of the mass is due to the cooling of the mercury to a point of solidification. Platinum is excepted from the list of metals classed in this behavior. This theory seems satisfactory to Dr. Flagg and his coadjutors, however it may fail to satisfy the reader. We cannot feel that the reasoning of this section of the book is very philosophic; but the experimentation as to edge strength by the various combinations of metals in varying proportions is quite fascinating to the metallurgic student. This section is evidently the embodyment of a great deal of observation. Dr. Flagg settles on silver and tin as the proper main ingredients in all alloys for amalgams, and silver and tin in the proportions of about sixty parts of the former to forty of the latter, with such proportions of gold and copper, with possibly platinum, and more favorably zinc, as modify setting, shrinkage, discoloration, etc. Large percentages of tin he asserts, as the result of long observation, are detrimental, causing "bulging" and "crevicing" from the spheroidal tendency, though why tin should have this tendency in a greater degree than any other metal it is difficult to understand, nor does the book explain it. The usefulness and preservative character of silver is, he thinks, largely due to the action of sulphur on this metal, forming sulphides. Copper is classed as an exceedingly useful, even essential, constituant in alloys, and especially of the "submarine" which is one of the many varieties of Dr. Flagg. He claims also that pulps are less apt to die with a copper-amalgam filling in proximity, than of an alloy from which this metal is excluded. It is claimed that copper in many cases tends to make a whiter filling, and also, which is more important, diminishes shrinkage. Gold is, according to Dr. Flagg, an "undetermined element," although its effect in controlling shrinkage is proven. Contradictory opinions are expressed by authorities quoted in this book as to the effect of gold in modifying the setting of amalgam; but Dr. Flagg concludes from an "immense experience" that "gold, more than any other metal, in proportion to the amount required, diminishes shrinkage, increase rapidity of setting, imparts fine grained plasticity, controls maintenance of color, and secures desirable edge-strength to amalgams." He thinks the quantity desirable as yet undetermined, but infers it should be from five to seven per cent. Antimony controls shrinkage, but makes an alloy dirty to work. Zinc, in the proportion of "silver sixty, tin forty, zinc one to one and a half parts, seems to control shrinkage perfectly. The working of such amalgams is described as 'peculiar,' but good." Why Dr. Flagg should warn us against zinc, as related to Cadmium it is difficult to understand. But on Cadmium he is severe, as producing disastrous results, destroying pulps, and doing other damage. Platinum is more severely treated than we had expected, as a useless component of alloys for amalgam. Curiously enough, in this department of this book the assertion is made, that careful analysis of some of the "gold and platinum alloys" in the market demonstrated that they contained none of this metal. The question is raised if the presence of platinum is not positively detrimental, and the statement is made—Dr. Flagg is famous for "statements,"—"that one part of zinc is worth a dozen times more than a dozen parts, or any other number of parts, of platinum."

Article VI is on the working of amalgam alloys, and well defined directions are given as to the best method of proceeding in alloying high with low fusing metals. The directions are as might be expected, different from those usuallp given, and seem reasonable. For it is certainly better to melt those metals which fuse at a high temperature by the aid of those which, melting low, readily alloy with them, rather than risk oxydation and loss of those capable of oxydation. A worker of a "celebrated" amalgam lately said to the writer that zinc was so difficult to alloy with other metals as to make it practically impossible to melt it with these. A very little experimentation with tin would have taught him better. A statement of some interest is that the cuttings of alloys for amalgams work better, take up mercury, and are altogether more satisfactory if exposed for some time then when freshly cut. This is in contradiction to the generally received opinion and also of the directions which accompany some alloys. And further it is laid down as a law that the better alloys take more mercury than those which are of low grade. Some valuable directions are given in the close of this chapter on qualitative testing of amalgam alloys, which must greatly interest the dental metallurgist.

We have not time, and it would extend this article unreasonably, to more than notice Article VII, entitled "Tests for Amalgams." These are classed as "Quality tests," in which the assertion is made, but no reasons given for it, that double-distilled mercury is unessential; the ordinary mercury of the shops being, so Dr. Flagg avers, good enough. It seems to the writer however that the idea of using the same relative quantity of mercury in all amalgams, irrespective of their other characteristics, and in the face of the statement made elsewhere in the book that the tin amalgam requires much less mercury than those he designates as "fine" is unjust to such alloys. If the spheroidal tendency is so marked in the largely tin alloys, and they take less mercury, it is unfair to them to treat these as the others which take much more mercury. It may be fair to infer that if those alloys condemned by the "quality test" of Dr. Flagg had been mixed with less mercury they might have had much greater "edge-strength."

The "shrinkage test" seems to simply prove that *all amalgams shrink*, that some shrink less than others, that many shrink irregularly, and that the indefiniteness about all this is of not much importance, since shrinkage is a valuable property, at least sometimes. Tin, Dr. Flagg insists is the "heavy shrinker." In the "setting test," the quantity of mercury best adapted to the proper working of the alloy is ascertained and used, abandoning the "*dry-mix*" and the use of warm instruments. In the "*color test*," the statement is made that an amalgam which will not discolor at all, should be looked on with suspicion, if the preservation of the tooth is the prime consideration. "Edge strength" is a phrase of Dr. Flagg's own invention, and he devised special apparatus for determining this. The "*tooth conserving test*" we are already familiar with.

Article VIII, on the "Preparation of Cavities," states that "bulging and crevicing" are the bug-bears of the amalgam filler," and that cavities should be decidedly larger within than without. "Leakage cannot be prevented; it comes through the soft tissue of the tooth itself," but such leakage,—this is the teaching of the "New Departure Corps,"—while damaging and ruinous in gold, is beneficial in amalgam. Yet we feel that in spite of all this we should prefer non-leakage, if possible. Article IX on the "Mixing of Amalgam" is an epitome of the now well known views of the writer on the questions of "working," "mortar-mixing," "plasticity" and "cold-working." This last, while not insisted on as making a better filling, the easy introduction is an offset to any of the objectionable features in soft amalgams largely containing mercury.

Much stress is laid on the "one admixture" doctrine. That is that the proper quantity of mercury should be mixed with the alloy and no more added. It is urged that as setting commences at once, any addition of mercury destroys the definiteness of the compound. The overheating by combining with too much mercury melts out certain of the ingredients in unknown quantities, while the overcooling by adding too much filling has the opposite effect; it chills the mercury in such wise as to prevent a given amount from doing its proper work of fusion." Dr. Flagg is here assuming that the theory as to the action of mercury on metals with which it unites is a definitely ascertained law, instead of a clever hypothesis. Low grade alloys, as he terms them—silver and tin—need only about thirty-seven to thirty-nine per cent. of mercury, while the higher grades of four metal alloys—silver, tin, copper and gold—about fifty per cent., "mortar-mixing" being essential; this is followed by a no less essential "palm-mixing," and according as this is done well or clumsily, it is the test of a plastic filler. It is a curious commentary on the whole subject of plastic filling that the worker of amalgam is to be graded as "skillful or unskillful in the working of this material," in proportion as he does this palm-mixing to suit Dr. Flagg's idea. Much delight is felt by the plastic worker when he hears the "cry" of the crepitation of the mass, almost akin one would imagine to the delight of the mother at hearing the cry of her new-born babe.

Article X is devoted to instruments, but as this paper has already exceeded its limits, we must pass this, and the chapter on "Insertion of Fillings." The remarks on the probable injury done to amalgam fillings by burnishing before they are fully set, are judicious, as it is probable much injury is done in this way, by cracking off thin edges. It is doubtful if any burnishing is useful, though careful finishing is important.

Article XII, on "local effects, systemic effects, and possibilities of amalgam" is sprightly reading, and in it

Dr. Flagg admits that he urges amalgam with "partizanship," but pluckily asserts that it is and has been used by the very men who have most loudly abused it. The makers of amalgams by the ton, all over the country, will not thank Dr. Flagg for the assertion that it is his object to give such information on the subject as shall "drive all ordinary materials from the market," unless duly labelled as oleomargerine ; and that "this arrangement will exclude the vast majority of amalgam alloys as at present manufactured and used. Some things in this chapter are good reading, but the Dr's enthusiasm carries him into "highfalutin" sometimes so decidedly as to suggest the thought that if he had read some paragraphs to a friend, kindly advice would have led to erasure of some things said. But in the main this chapter is one of the best in the book, and most of us will learn something from its perusal, on the subject of building up broken teeth.

Article XIII, on Gutta-percha, has little that was not known before, but some points in manipulation will be useful. But the reader will pause before accepting fully the statement on page 149 that "as the result of over fifteen years of careful observation, and with a basis of over two thousand replacements of gold with gutta-percha," the latter, "if properly used is *at least twice* as durable as gold, and that in *very soft teeth*, in selected places, it is fair to presume that it will preserve its tooth *at least three times as long as a well introduced gold filling*" (*!*) He is careful through, to say that this is an *assertion*, not an *opinion*.

It had been taught as we remember, that oxy-chlorides expanded, and that it was not safe to use these in very thin frail teeth. But Dr. Flagg's statement, in Article XIV is that they all shrink ; and further that as a filling material it is completely ignored by the plastic worker, he only using it for foundations, linings, etc. Oxy-sulphate of zinc is recommended as a capping material for exposed pulps as better from a therapeutic standpoint than anything in its direction. The zinc phosphates are energetically condemned and justly so, for surface filling, and he thinks that they have nothing to commend, them over, but much to put them below, the other zinc fillings. A nitrate of zinc is spoken of favorably, but, it is doubtful if it is better than the others.

Article XVIII, on Technicalities, is a resume of the preceding, with the nomenclature of the "New Departure." Sprightly reading; but the good Dr. gets on the war-path again fiercely.

We conclude, this is a somewhat valuable book, though it is difficult to get out of it the things one wants. To one who can read it with judgment, it will be serviceable. But we caution young men especially to be careful, just as careful of their gold fillings as ever. We have seen too much good, durable gold work, in soft teeth to abandon it! And we urge too on the attention of all, the statement of that renowned worker of amalgam, Dr. Clowes, that in proportion as one can fill with gold well he can manipulate amalgam well. It is like the training which gold plate work gives the mechanist—he can work plastic plates all the better for it. But Dr. Flagg's book will make converts, who will straightway rush into his ways, and with all carelessness use plastics, to be condemned; and then comes reaction. Our advice to the young in all these mixed fillings, is to mix them with *judiciousness.*

One fact which stands out in this book, as in Dr. Flagg's Dental Pathology and Therapeutics is worth pondering over. It is that a tooth with a dead pulp is treated as an invalid, ready to relapse into outright sickness at any time. So his methods are such as enable him to remove that which he uses as the root filling at any time. We have no time to go into this subject at present. Dr. Spencer Bates, F. R. S., of Plymouth, England, in 1875, at a meeting of the New York Odontological Society, in a paper read there, suggested the idea of filling roots of teeth with glycerine, and Dr. Flagg there said he "used to be particular in asserting that he was one of those *who went to the extreme end of*

*every canal.* In every tooth he laid this down as a fundamental law!

In this book as well as in his writings generally, evidencing the Doctor's characteristics, he goes, as he did in his root canals to the extreme. We feel shy of extremities. In following Dr. Flagg, we advise our young men again and again to be cautious.

